# Contribution of *Streptococcus mutans* to *Helicobacter pylori* colonisation in oral cavity and gastric tissue

**DOI:** 10.1038/s41598-020-69368-2

**Published:** 2020-07-27

**Authors:** Ryota Nomura, Tamami Kadota, Yuko Ogaya, Saaya Matayoshi, Naoki Iwashita, Rena Okawa, Kazuhiko Nakano

**Affiliations:** 1 Department of Pediatric Dentistry, Osaka University Graduate School of Dentistry, 1-8 Yamada-oka, Suita, Osaka 565-0871 Japan; 2 Department of Pharmacology, School of Veterinary Medicine, Azabu University, Sagamihara, Kanagawa Japan

**Keywords:** Microbiology, Bacteria, Bacterial pathogenesis

## Abstract

*Helicobacter pylori* is presumed to infect gastric tissue via the oral cavity in childhood, whereas risk factors for *H. pylori* infection in the oral cavity are unknown. In this study, we analysed the effects of *Streptococcus mutans*, a major cariogenic bacterial species, on *H. pylori* colonisation in the oral cavity, as well as gastric tissue. Rats in the weaning period were infected with *S. mutans* in the oral cavity, then fed a caries-inducing diet to facilitate *S. mutans* colonisation. One month after *S. mutans* infection, rats were infected with *H. pylori* in the oral cavity; rats were then euthanised at 1 month after *H. pylori* infection. *H. pylori* was detected in the oral cavities of rats infected with both *S. mutans* and *H. pylori*, but not in rats infected with *H. pylori* alone. In addition, *H. pylori* colonisation in the gastric tissue and typical gastrointestinal damage were observed in rats infected with both *S. mutans* and *H. pylori*. When *H. pylori* was co-cultured with in vitro biofilm formed by *S. mutans*, a large number of *H. pylori* bacteria invaded the biofilm formed by *S. mutans*. Our results suggest that *S. mutans* is involved in the establishment of *H. pylori* infection.

## Introduction

*Helicobacter pylori*, a helix-shaped gram-negative microaerophilic bacterium, is a major causative agent of gastric cancer and gastric ulcers^1^. More than half of the world’s population is infected with *H. pylori*^2^, which is presumably acquired mainly via the oral cavity in childhood^3,4^. Molecular biological techniques have reportedly revealed *H. pylori* in oral specimens^5–7^. The presence of *H. pylori* in the oral cavity has been related to the detection of *H. pylori* in the gastric tissue^8^. However, details regarding risk factors of *H. pylori* infection in the oral cavity have not been clarified, which may explain the current difficulty in elimination of *H. pylori* infection.

*Streptococcus mutans*, a gram-positive facultative anaerobe, is a major causative pathogen of dental caries^9^. *S. mutans* is acquired in the oral cavity during early childhood, mainly via mother-to-child transmission^10^. The aetiology of dental caries caused by *S. mutans* was clarified in the early 1960s^11^; *S. mutans* metabolises sucrose to form a biofilm on the tooth surface, followed by demineralisation of the tooth. Nevertheless, eradication of *S. mutans* from the oral cavity and dental caries remains difficult^12^.

Some epidemiological studies have revealed that patients with dental caries or poor oral hygiene were more likely to harbour *H. pylori* in oral cavity or gastric tissue^13,14^. These findings suggest that the presence of cariogenic bacteria is involved in infection of the oral cavity with *H. pylori*. To the best of our knowledge, no clear evidence has been obtained regarding the effects of *S. mutans* on *H. pylori* infection in an animal model. In the present study, we hypothesised that *S. mutans* colonisation in the oral cavity may be involved in *H. pylori* colonisation in both oral cavity and gastric tissue. Therefore, we constructed a rat co-infection model with *S. mutans* and *H. pylori*. Using this model, we analysed the effects of *S. mutans* on *H. pylori* colonisation in the oral cavity and gastric tissue.

## Results

### Dental caries status and detection of bacteria in the rat oral cavity

**Figure 1:**
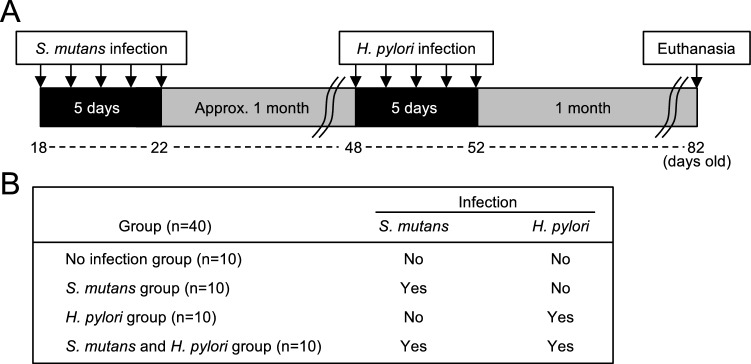
Schematic of rat model experimental protocol. (**A**) Experimental schedule. (**B**) Groups of rats in this experiment.

**Figure 2:**
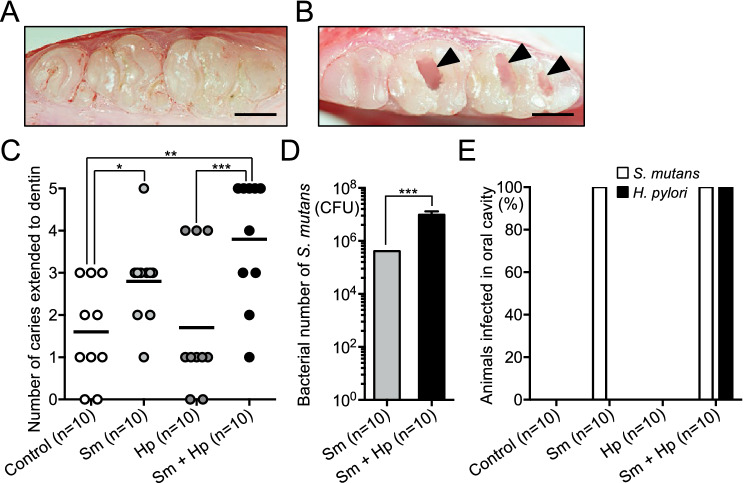
Dental caries status and detection of bacteria in the rat oral cavity. Representative images of the teeth of rats (**A**) without and (**B**) with dental caries. Arrowheads indicate dental caries. Bars = 500 μm. (**C**) Numbers of teeth with dental caries. Each closed circle represents the number of dental caries for a single rat. Horizontal bars indicate mean values for respective groups. (**D**) Numbers of *S. mutans* bacteria. (**E**) Detection rates of bacteria in the oral cavity. Significant differences were observed, using analysis of variance with Bonferroni correction (*P < 0.05, **P < 0.01 and ***P < 0.001). Sm, *S. mutans*; Hp, *H. pylori*.

In our experimental procedure, rats were fed a caries-inducing diet containing 56% sucrose (CLEA Japan, Osaka, Japan) throughout the experiment to induce dental caries^15^; they were divided into four groups, depending on the presence or absence of infection with *S. mutans* and *H. pylori* (Fig. 1A,B). Rats were euthanised at 82 days of age and dental caries status was evaluated using excised maxillary and mandibular bones. Representative images of teeth from rats without and with dental caries are shown in Fig. 2A,B. Mean numbers of dental caries were significantly higher in rats that had been infected with *S. mutans* than in rats that had not been infected with *S. mutans*, regardless of *H. pylori* infection (*P* < 0.05) (Fig. 2C). The number of *S. mutans* isolated from the mandibular bone was significantly higher in rats infected with both *S. mutans* and *H. pylori*, compared with rats infected with *S. mutans* alone (*P* < 0.001) (Fig. 2D). Although the most severe dental caries were observed in rats that had been infected with both *S. mutans* and *H. pylori*, average number of dental caries did not significantly differ compared with rats that had been infected with *S. mutans*.

Polymerase chain reaction (PCR) and nested PCR were performed to detect *S. mutans* and *H. pylori*, respectively, in dental plaque specimens collected from rats aged 55–82 days; PCR primers are shown in Table 1. *S. mutans* was detected in the oral cavities of all rats that had been infected with *S. mutans* (Fig. 2E), whereas no *S. mutans* was detected in rats that had not been infected with *S. mutans*. Notably, *H. pylori* was not detected in rats that had been infected with *H. pylori* alone; however, *H. pylori* was detected in all rats that had been infected with both *S. mutans* and *H. pylori*, which indicated that *S. mutans* was essential for *H. pylori* colonisation.

**Table 1:** Polymerase chain reaction primers used in the present study.

Specific primer set	Sequence (5′-3′)	Size (bp)	References
**Detection of* S. mutans***			
MKD-F	GGC ACC ACA ACA TTG GGA AGC TCA GTT	433	^16^
MKD-R	GGA ATG GCC GCT AAG TCA ACA GGA T		
**Detection of* H. pylori***			
** First step PCR**			
*ureA*-aF	ATG AAA CTC ACC CCA AAA GA	488	^17^
*ureA*-bR	CCG AAA GTT TTT TCT CTG TCA AAG TCT A		
** Second step PCR**			
*ureA*-bF	AAA CGC AAA GAA AAA GGC ATT AA	383	^17^
*ureA*-aR	TTC ACT TCA AAG AAA TGG AAG TGT GA		

### Histopathological evaluation of rat gastric tissue

**Figure 3:**
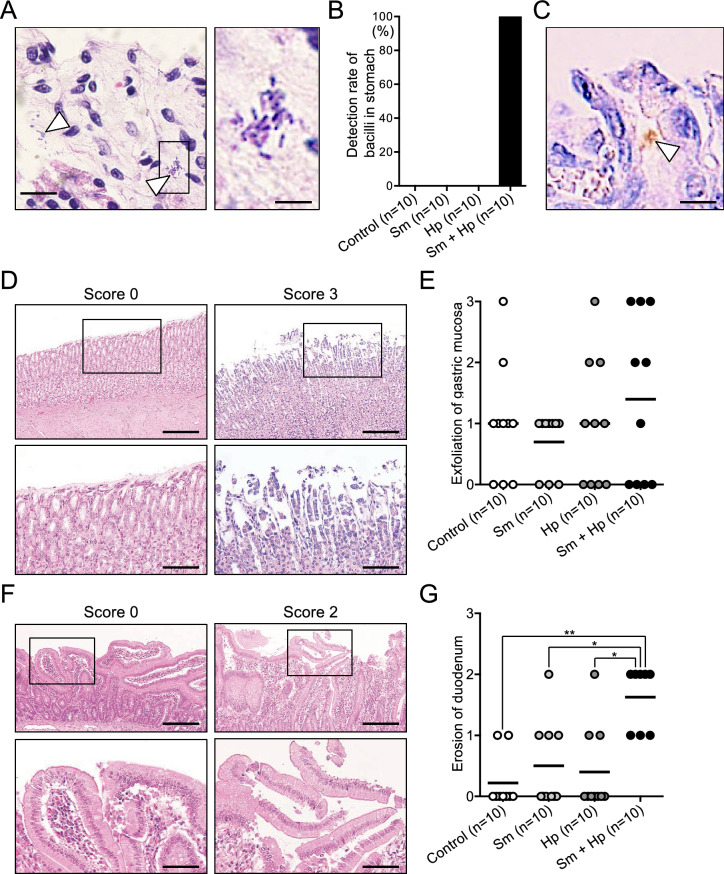
Bacterial detection and histopathological evaluation in gastric tissue. (**A**) Representative images of histopathological features observed in HE-stained tissue. Right panel shows high-magnification image of the box on the left image. Arrowheads indicate bacilli. Bars = 50 μm (left panel) and 10 μm (right panel). (**B**) Detection rates of bacilli in gastric tissue. (**C**) Representative image of immunohistochemical staining findings, using an *H. pylori*-specific antibody. Arrowhead indicates positive *H. pylori*-specific antibody staining. Bar = 10 μm. (**D**) Representative images of gastric mucosal exfoliation with different histopathological scores, as determined by HE staining. Lower panels show high-magnification images of the boxes on upper images. Bars = 300 μm (upper panels) and 100 μm (lower panels). (**E**) Histopathological scores of gastric mucosal exfoliation. Each closed circle represents the gastric mucosal exfoliation score for a single rat. Horizontal bars indicate mean values for respective groups. (**F**) Representative images of duodenal erosion with different histopathological scores, as determined by HE staining. Lower panels show high-magnification images of the boxes on upper images. Bars = 300 μm (upper panels) and 100 μm (lower panels). (**G**) Histopathological scores of duodenal erosion. Each closed circle represents the duodenal erosion score for a single rat. Horizontal bars indicate mean values for respective groups. Significant differences were observed, using analysis of variance with Bonferroni correction (*P < 0.05 and **P < 0.01). Sm, *S. mutans*; Hp, *H. pylori*.

*Helicobacter pylori* infection in excised rat gastric tissues was analysed by histopathological evaluation. In all rats that had been infected with both *S. mutans* and *H. pylori*, invasion of bacilli into gastric tissue was confirmed by haematoxylin and eosin (HE) staining (Fig. 3A,B); immunostaining analysis confirmed that these bacilli were *H. pylori* (Fig. 3C). However, no bacilli were detected in other groups, including rats infected with *H. pylori* alone. Subsequently, qualitative analysis of HE-stained stomach and duodenum histopathological findings was performed. Representative images of gastric mucosal exfoliation are shown in Fig. 3D. The mean gastric mucosal exfoliation score was highest in rats infected with both *S. mutans* and *H. pylori* (Fig. 3E), although this score did not significantly differ from the scores of other groups. In addition, representative images of duodenal erosion are shown in Fig. 3F. The duodenal erosion score was significantly higher in rats infected with both *S. mutans* and *H. pylori* than in other groups (*P* < 0.05) (Fig. 3G). The scores of other histopathological findings did not significantly differ among the groups (see Supplementary Figure 1 online).

### In vitro bacterial growth and biofilm assays with co-cultured *S. mutans* and *H. pylori*

**Figure 4:**
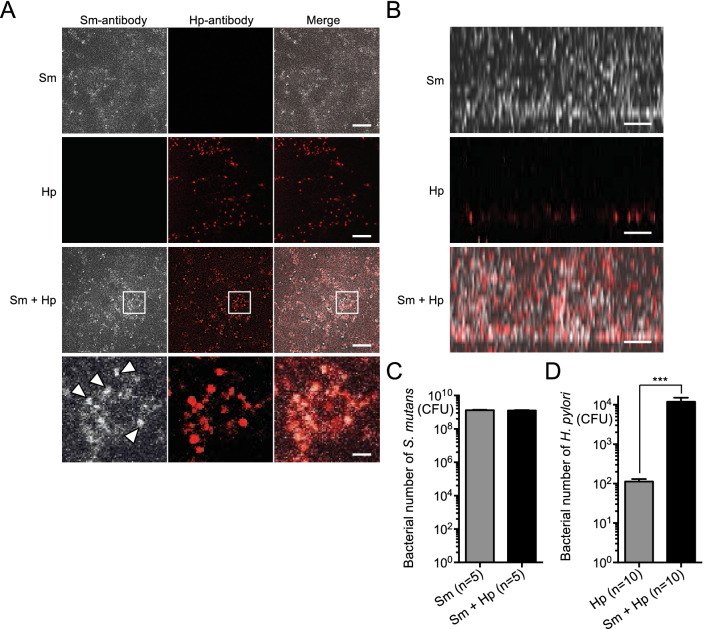
Biofilm formation by co-cultured *S. mutans* and *H. pylori*. (**A**) Representative two-dimensional images of biofilm, captured using confocal scanning laser microscopy. Bars = 15 μm. Lower panels of co-cultured *S. mutans* and *H. pylori* show high magnification images of square parts of upper panel. Arrowheads indicate dense growth of *S. mutans*. Bars = 300 nm. (**B**) Representative enlarged images of biofilm thickness, captured using confocal scanning laser microscopy. *S. mutans* and *H. pylori* cells are stained white and red, respectively. Bars = 10 μm. (**C**) Numbers of *S. mutans* bacteria. (**D**) Numbers of *H. pylori* bacteria. Significant differences were observed, using analysis of variance with Bonferroni correction (***P < 0.001). Sm, *S. mutans*; Hp, *H. pylori*.

In vitro assays were performed to analyse the colonisation of *H. pylori* in the presence of *S. mutans*. Notably, the presence of the culture supernatant of *S. mutans* did not affect the growth of *H. pylori* (Supplementary Figure 2). A subsequent in vitro biofilm assay was performed using both *S. mutans* and *H. pylori*. *S. mutans* is known to form a biofilm with high adhesiveness in the presence of sucrose, and in vitro experimental systems for biofilm formation are widely used^18–20^. In our biofilm system, *S. mutans* is grown in a medium supplemented with sucrose on a cover glass or polystyrene plate, which are regarded as simulated tooth surfaces, and incubated at 37 °C for 18 h. To confirm that the presence of *S. mutans* is required for *H. pylori* colonisation in the oral cavity, *S. mutans* and *H. pylori* were co-cultured using the in vitro biofilm assay. *H. pylori* was observed to form flat layers in two-dimensional images, regardless of the presence of *S. mutans* (Fig. 4A). Notably, *H. pylori* was especially localised in dense areas of *S. mutans* growth. In addition, three-dimensional imaging revealed that the location of *H. pylori* in the biofilm was dependent upon the presence or absence of *S. mutans* (Fig. 4B, Supplementary Figure 3). When cultured without *S. mutans*, *H. pylori* was found adhered to the surface of the plate in a single monolayer. In contrast, when *S. mutans* and *H. pylori* were co-cultured, *H. pylori* was found distributed throughout the biofilm formed by *S. mutans*. There was no difference in the number of *S. mutans* between cultures of *S. mutans* alone and cultures containing both *S. mutans* and *H. pylori* (Fig. 4C). In contrast, the number of *H. pylori* was significantly higher in cultures containing both *S. mutans* and *H. pylori* (1.2 × 10^4^ colony-forming units [CFUs]) than in cultures of *H. pylori* alone (1.1 × 10^2^ CFUs) (*P* < 0.001) (Fig. 4D). In cultures containing both *S. mutans* and *H. pylori*, 1 CFU of *H. pylori* was present for approximately 1 × 10^5^ CFUs of *S. mutans*.

## Discussion

*H. pylori* is presumably transmitted through the oral cavity in childhood^21^, then resides in the human body unless eradication therapy is administered. Although several epidemiological studies have shown that *H. pylori* colonisation in the oral cavity is associated with its presence in gastric tissue^22–24^, this relationship remains controversial. In the present study, the presence of *H. pylori* in rat oral cavity was positively correlated with its presence in gastric tissue. In addition, the results showed that cariogenic bacteria are involved in *H. pylori* colonisation in these organs.

Epidemiological studies have shown that individuals with dental caries are more susceptible to *H. pylori* infection^13,14^. In contrast, a clear relationship between dental caries and *H. pylori* colonisation has not been demonstrated in an animal model, because only a few research groups can successfully colonise cariogenic bacteria in the oral cavity of animal models. In humans, *S. mutans* mainly infects infants between 19 and 31 months of age^10^. Similarly, it has been shown that administration of *S. mutans* to 18-day-old rats can establish *S. mutans* colonisation in the oral cavity^15^; moreover, a dental caries model can be induced by feeding a caries-inducing diet containing 56% sucrose for 1–2 months. Using a rat model, we orally administered *H. pylori* and achieved successful *H. pylori* colonisation.

There was no significant difference in the severity of dental caries between rats infected with *S. mutans* and those infected with *H. pylori*. In contrast, previous epidemiological studies showed that patients with *H. pylori* in dental plaque demonstrated a significantly higher occurrence of dental caries, compared with patients who did not exhibit *H. pylori* in dental plaque^13,25^; those results suggested that *H. pylori* is involved in dental caries progression. In addition, the present study evaluated the dental caries status of rats infected with *S. mutans* at the age of 82 days. We previously analysed the occurrence of dental caries in rats in a time-dependent manner^26^, which revealed that most rats infected with *S. mutans* had mild dental caries localised to hard tissues, despite an age greater than 90 days. This occurrence of mild dental caries may have resulted in the absence of significant differences in the numbers of dental caries between *S. mutans*- and *H. pylori*-infected groups. To more comprehensively compare the severity of dental caries between rats with *S. mutans* infection and rats with *H. pylori* infection, rats of different ages should be included in future analyses of dental caries progression. In addition, X-ray photographs of the teeth may be needed for more accurate diagnoses, whereas we evaluated the presence or absence of dental caries using a sterilised 27G needle under a stereomicroscope.

In the present study, most severe dental caries were observed in rats that had been infected with both *S. mutans* and *H. pylori*. A recent in vitro study revealed that *H. pylori* could change the balance of oral biofilm induced by oral streptococci^27^; the authors of that study suggested that *H. pylori* may affect signalling among oral streptococci involved in biofilm formation, and that *H. pylori* could create an advantageous environment for *S. mutans*. Indeed, higher numbers of *S. mutans* were isolated from excised mandibular bones of rats infected with both *S. mutans* and *H. pylori*, compared with mandibular bone from rats infected with *S. mutans* alone. Therefore, interactions between *H. pylori* and oral bacteria may affect both colonisation by *H. pylori* and the pathogenicity of oral bacteria. Although the main purpose of the present study was to examine the effects of *S. mutans* on *H. pylori* colonisation in oral and gastric tissue, the findings regarding effects of *H. pylori* on cariogenic properties of *S. mutans* provide important insights for future research.

In the present study, dental caries were induced in some rats which were not infected with any bacteria. This result is consistent with the findings of a previous study, in which mild dental caries occurred in some rats which were not infected with caries-causing bacteria^26^. In addition, oral bacteria associated with severe dental caries were detected in periapical lesions of uninfected rats which had undergone artificial exposure of dental pulp via endodontic instrumentation^28^. Therefore, commensal bacteria in the rat oral cavity of rats may also cause mild dental caries.

Recently, inflamed dental pulp obtained from human patients has been considered a possible source of *H. pylori* infection^7,17^. Therefore, we analysed the presence of *H. pylori* in pulp tissue collected by using a sterilised dental handpiece and diamond point. Our results revealed that *H. pylori* was not present in the dental pulp, probably because few severe dental caries extended to dental pulp in our rat model.

Gastric mucosal injury and duodenal erosion are regarded as the major symptoms of *H. pylori* gastritis^29,30^. In the present study, rats that had been infected with both *S. mutans* and *H. pylori* exhibited greater damage to the digestive tract, compared with rats that had not been infected with *H. pylori*. However, these rats did not exhibit severe gastrointestinal diseases, such as gastric cancer. Notably, the combination of *H. pylori* infection with other risk factors (e.g., excessive salt and smoking) is closely related to the occurrence of serious gastrointestinal diseases^31^. Thus, to induce severe gastrointestinal symptoms in a rat model, these risk factors may need to be combined with the bacterial infection approach used in the present study.

A recent study showed that *H. pylori* was unsuitable for growth assays involving co-cultivation with oral streptococci, due to the stringent growth requirements of *H. pylori*^27^. In that study, the *H. pylori* culture supernatant was added to broth cultures of oral streptococci, which allowed analysis of the effect of *H. pylori* on the growth of oral streptococci. In the present study, we used a modified method to analyse the growth of *H. pylori* in the presence of *S. mutans* culture supernatant. Our results showed that *S. mutans* did not affect the growth of *H. pylori*. Another previous study found that *H. pylori* growth inhibition depends on the *H. pylori* strains or the oral bacterial species used in a particular assay^32^. Thus, it may be necessary to further analyse the influences of specific *S. mutans* and *H. pylori* strains on the growth of *H. pylori*.

The *S. mutans *in vitro biofilm assay has been performed in prior studies^19,20^. When *H. pylori* alone was grown in the in vitro biofilm assay, only a single layer of *H. pylori* was observed, which suggested that a small amount of *H. pylori* might adhere to the tooth surface. In contrast, when both *S. mutans* and *H. pylori* were grown in biofilm, multilayer *H. pylori* growth was observed in biofilm formed by *S. mutans*. The results suggested that *H. pylori* can penetrate dental plaque that forms on the tooth surface; however, *S. mutans* is predominant in biofilm that forms in the presence of sucrose, and the amount of *H. pylori* is considerably smaller than the amount of *S. mutans*.

Bacteria generally interact with each other in oral biofilms to facilitate survival in the oral environment^33^. In addition, the aggregation of multiple bacterial species aids each bacteria in colonisation of the oral cavity^34^. In our biofilm assay, *H. pylori* was colocalised with *S. mutans*. Therefore, *H. pylori* presumably utilises the biofilm formed by *S. mutans* to survive and colonise the oral cavity.

Despite its useful findings, some questions remain unresolved in this study. For example, it is unclear how a large number of *H. pylori* bacteria can enter biofilm formed by *S. mutans*. To elucidate the mechanism of *H. pylori* invasion of biofilm, molecular biological analyses are necessary; these analyses should focus on pathogenic proteins or signalling systems of both *H. pylori* and *S. mutans*. In addition, it remains unknown whether *S. mutans* alone can support *H. pylori* colonisation in the oral cavity. Thus, analyses using the rat model of *H. pylori* co-infection should be performed with other oral bacteria. Furthermore, it remains unclear whether the presence of *S. mutans* alone or in combination with the occurrence of dental caries is important for *H. pylori* colonisation. Thus, future studies should include comparisons of rat models with *S. mutans* colonisation in the oral cavity, with or without severe dental caries, to determine the relationships of these specific aspects with *H. pylori* colonisation.

In summary, we demonstrated that the presence of *S. mutans* in the oral cavity was able to support *H. pylori* colonisation in both oral cavity and gastric tissue. In addition, we found that *H. pylori* was able to invade biofilm formed by *S. mutans*. These results suggest that prevention of *S. mutans* infection in childhood, as well as the establishment of preventive habits to avoid *S. mutans* colonisation in the oral cavity (e.g., good oral hygiene and sucrose restriction) may be effective for prevention of *H. pylori* infection.

## Methods

### Bacterial strains and growth conditions

*S. mutans* strain MT8148R, a streptomycin-resistant substrain of MT8148^35^, was grown from our laboratory stock. MT8148R was cultured on Mitis Salivarius agar (Difco Laboratories, Detroit, MI, USA) plates containing bacitracin (0.2 U/ml; Sigma Chemical Co., St. Louis, MO, USA) and 15% sucrose (i.e., MSB agar) containing 1500 μg/ml streptomycin at 37 °C for 2 days under 95% N_2_ and 5% CO_2_ condition. For routine growth, *S. mutans* was grown in brain heart infusion broth (Difco Laboratories) containing 1500 μg/ml streptomycin at 37 °C for 18 h. *H. pylori* strain J99 (ATCC 700824) was purchased from Summit Pharmaceuticals International Corporation (Tokyo, Japan). *H. pylori* was cultured using blood agar plates (Becton Dickinson, Franklin Lakes, NJ, USA) at 37 °C for 3 days under microaerophilic conditions. Colonies were then inoculated in 10 ml brucella broth (Becton Dickinson) supplemented with 1 ml horse serum, and incubated at 37 °C for 3–5 days. Cultured *S. mutans* and *H. pylori* strains in their respective broths were harvested and washed with sterile saline, then used in the following experiments.

### Rat model experimental protocol

The experimental protocol by which rats were infected with *H. pylori* strain J99 and/or *S. mutans* MT8148R, then stimulated to form dental caries, is shown in Fig. 1A. Dental caries were induced using a previously described method^15^. Briefly, 40 male Sprague–Dawley rats, aged 15 to 18 days, were fed a normal diet CE-2 (CLEA Japan) containing tetracycline (4 mg/g) and given water containing penicillin G (4000 U/ml), prior to the establishment of bacterial colonisation in the oral cavity. All rats were then fed a caries-inducing diet containing 56% sucrose (CLEA Japan) until the end of the experiment. At 18 days of age, oral infection of *S. mutans* (1 × 10^8^ CFUs in sterile saline) was performed in 20 rats, once per day for 5 days to establish *S. mutans* colonisation in the oral cavity; the remaining 20 rats did not receive *S. mutans*. One week after infection, dental plaques were collected from the oral cavity of each rat using a sterilised cotton swab; plaque samples were then seeded in MSB agar containing 1500 μg/ml streptomycin to confirm that *S. mutans* colonisation had been successfully established in the oral cavity.

Twenty rats (10 *S. mutans*-infected rats and 10 previously uninfected rats) were infected with *H. pylori* at 48 days of age; the rats were divided into four groups according to the presence or absence of infection with *S. mutans* and *H. pylori* (Fig. 1B). For *H. pylori* infection, oral infection of *H. pylori* (1.5 × 10^6^ CFUs in sterile saline) was performed for 5 consecutive days. Dental plaque was collected once per week to assess *H. pylori* colonisation in the oral cavity, beginning 3 days after *H. pylori* infection and continuing until rats were euthanised. One month after *H. pylori* infection, rats were euthanised; maxillary and mandibular bones were then excised and used for detection of bacteria and evaluation of dental caries. The presence or absence of dental caries was determined by a dentist, who observed the occlusal surfaces of right maxillary and mandibular molar teeth (six teeth per rat) using a sterilised 27G needle (0.4 mm diameter) (Terumo Co., Tokyo, Japan) under a stereomicroscope. In addition, stomach and duodenum were excised for use in histological evaluation.

### Detection of bacteria from the oral cavity

Recovery of *S. mutans* strain from mandibular bone was evaluated by using a previously described method^36^. After rats had been euthanised, excised mandibular bones were placed in sterile saline and bacteria were separated from the bones by sonication. The resulting bacterial suspension was serially diluted with sterile saline and cultured on MSB agar plates containing 1500 μg/ml streptomycin. After the agar plates had been incubated at 37 °C for 48 h, the numbers of colonies on the agar plates were counted to determine the numbers of *S. mutans* present in the mandibular bones.

Bacterial DNA was extracted from dental plaque samples or excised maxillary and mandibular bones in sterile saline after sonication. *S. mutans* detection was performed by PCR with *S. mutans*-specific primers (Table 1). *H. pylori* detection was performed by nested PCR using previously described *H. pylori*-specific primers^17^. Briefly, first-step PCR was performed using primers *ureA*-aF and *ureA*-bR; second-step PCR was performed with the first PCR product as a template, using primers *ureA*-bF and *ureA*-aR. All PCR products were amplified using *Takara Ex Taq* (Takara Bio. Inc., Otsu, Japan), then visualised by electrophoresis in a 1.5% agarose gel.

### Histopathological evaluation of gastric tissue

All gastric and duodenal tissues were removed from each euthanised rat. The tissues were fixed in 10% neutral buffered formalin solution (Fujifilm Wako Pure Chemical Corporation, Tokyo, Japan), then embedded in paraffin and cut into 3-μm sections. These sections were subjected to HE staining, followed by evaluation of pathological features in all sampled gastric and duodenal tissues. Histopathological features were evaluated by scoring as follows: 0 (none), 1 (mild), 2 (moderate), and 3 (severe), in accordance with a previously published method with some modification^37^. Scoring was performed in a double-blinded manner by a pathologist (Sept. Sapie Co., Ltd, Tokyo, Japan).

Immunohistochemical staining of sections of stomach and duodenum was performed using a Vectastain ABC kit (Vector Laboratories, Burlingame, CA, USA), in accordance with the manufacturer’s instructions. First, sections were blocked with 3% H_2_O_2_ and 2.5% horse serum. Then, sections were incubated with anti-*H. pylori* antibody (Thermo Fisher Scientific, Waltham, MA, USA; diluted 1:100 with phosphate-buffered saline [PBS]) at 4 °C for 12 h. Subsequently, sections were incubated with secondary antibody from the kit at room temperature for 30 min. In addition, counterstaining was performed with haematoxylin solution.

### Bacterial growth of *H. pylori*

Bacterial growth of *H. pylori* was assessed using a previously described method^27^, with some modifications. *H. pylori* colonies were suspended in brucella broth supplemented with horse serum, then grown to an OD_550_ of 0.2. The culture supernatant of 1 × 10^7^ CFUs of *S. mutans* filtered by a 0.45 μm filter was added to the bacterial suspension. The growth activity of *H. pylori* was analysed by culturing the bacterial suspension at 37 °C under microaerophilic conditions and measuring the OD_550_ value at 12-h intervals. Data were recorded as the average of three independent analyses.

### Biofilm assay

Bacterial suspensions of *S. mutans* and *H. pylori* strains were adjusted to 1.0 × 10^7^ CFUs/ml in BHI broth containing 1% sucrose. Then, 200 µl of the suspensions were added to a chambered cover glass system (CultureWell, Grace Bio Labs, Bend, OR, USA) and incubated at 37 °C for 18 h under microaerophilic conditions. Non-attached bacterial cells were washed with PBS, while adherent cells were fixed with 3% paraformaldehyde (Wako Pure Chemical Industries, Osaka, Japan) for 10 min. For *S. mutans* staining, rabbit anti-PA serum^38^ was used as primary antibody and Alexa Fluor 633-conjugated goat anti-rabbit immunoglobulin G (Molecular Probes, Life Technologies Co., Eugene, OR, USA) was used as secondary antibody. For *H. pylori* staining, rabbit anti-*H. pylori* antibody (Thermo Fisher Scientific) was used as primary antibody and Alexa Fluor 533-conjugated goat anti-rabbit immunoglobulin G (Molecular Probes, Life Technologies Co.) was used as secondary antibody. Each antibody was diluted 1:500 in PBS containing 0.5% bovine serum albumin, then incubated with fixed cells for 30 min at room temperature. The chambered cover glass system was washed with PBS, before and after incubation with each antibody. Biofilms were observed by confocal scanning laser microscopy using a TCS-SP5 microscope (Leica Microsystems GmbH, Wetzlar, Germany), as well as a DMI6000 B fluorescence microscope (Leica Microsystems GmbH) and a 63 × oil immersion objective. The mean volume of *H. pylori* contained in the biofilm was determined by analysis of 10 separate confocal images in each group, using ImageJ software (National Institutes of Health, Bethesda, MD, USA).

To determine the number of *S. mutans* bacteria present in the biofilm, the formed biofilm was washed with PBS and removed by pipetting. The collected and serially diluted bacterial suspension was then cultured on MSB agar plates at 37 °C for 2 days. Subsequently, the number of *S. mutans* in each biofilm was calculated by counting the number of colonies on the corresponding agar plate. Data were recorded as the average of five independent analyses. The number of *H. pylori* was quantified by confocal scanning laser microscopy. The numbers of *H. pylori* colonies were initially counted in two-dimensional images. The number of *H. pylori* contained in the biofilm was then calculated by multiplying the number of colonies counted by the thickness of each image. Data were recorded as the average value of 10 separate images.

### Statistical analysis

Statistical analyses were performed using GraphPad Prism 6 (GraphPad Software Inc., La Jolla, CA, USA). Intergroup differences were compared using analysis of variance (ANOVA). Bonferroni correction was used for post hoc analyses. Differences with *P* < 0.05 were considered statistically significant.

### Ethical approval

All rats were treated humanely, in accordance with the guidelines of the National Institutes of Health and the AERI-BBRI Animal Care and Use Committee. All animal experiments were approved by the Institutional Animal Care and Use Committee of Osaka University Graduate School of Dentistry (Approval No. 29-031-0).

## Supplementary information

Supplementary information

## Data Availability

All data generated or analysed during this study are included in this published article (and its Supplementary Information files).
