# Supplementary material for: Contribution of Streptococcus mutans to Helicobacter pylori colonisation in oral cavity and gastric tissue
Source: Sci Rep. 2020 Jul 27;10:12540. doi: 10.1038/s41598-020-69368-2 (PMC7385622; doi:10.1038/s41598-020-69368-2)
Supplement: Supplementary file 1 — Supplementary information [file 41598_2020_69368_MOESM1_ESM.pdf]

Contribution of *Streptococcus mutans* to *Helicobacter pylori* colonisation in oral cavity and gastric tissue

Ryota Nomura, Tamami Kadota, Yuko Ogaya, Saaya Matayoshi, Naoki Iwashita, Rena Okawa, Kazuhiko Nakano

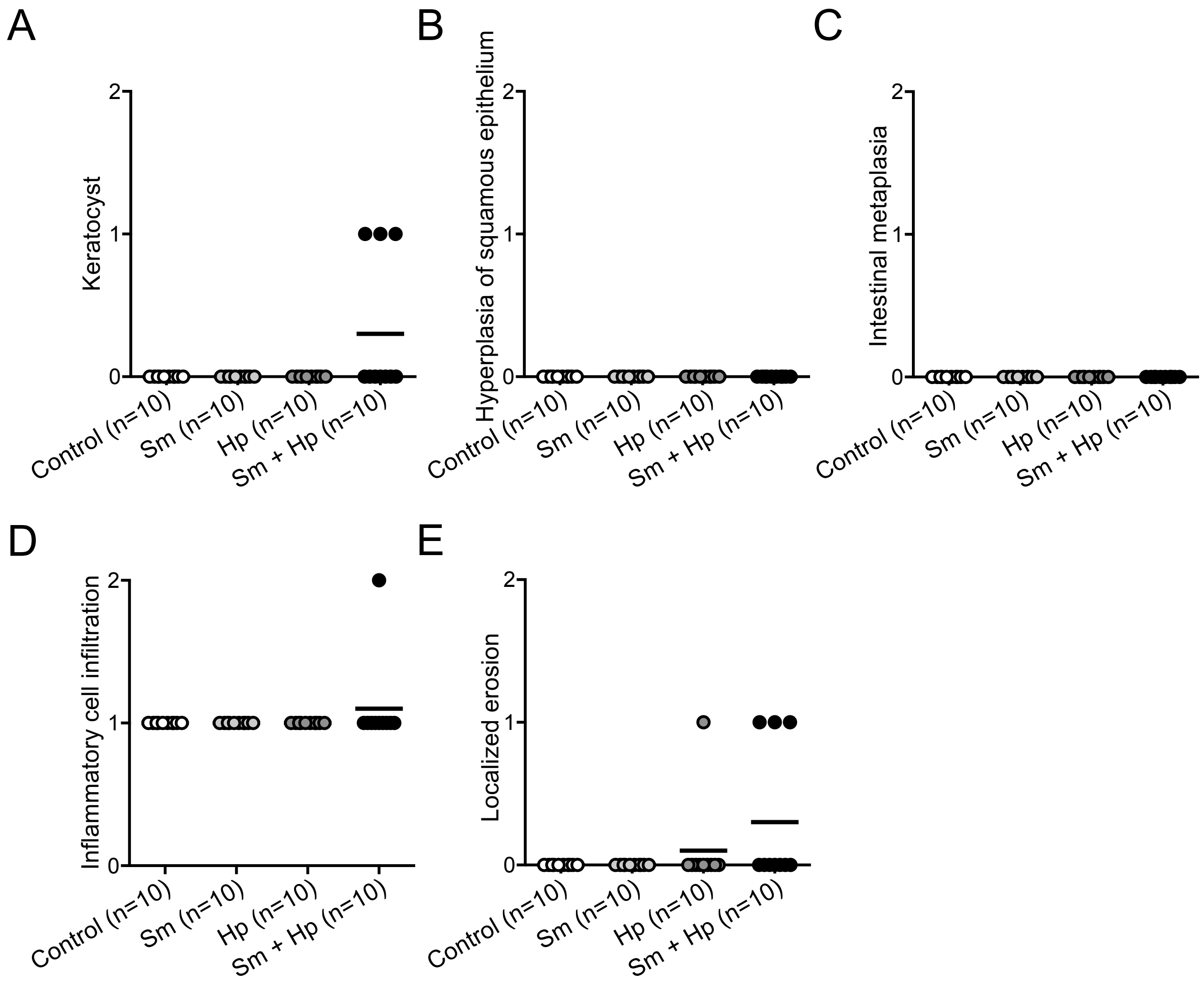

Supplementary Figure 1 Histopathological scores of (a) keratocyst, (b) hyperplasia of squamous epithelium, (c) intestinal metaplasia, (d) inflammatory cell infiltration, and (e) localized erosion. Each closed circle represents the deflection of gastric mucosa for each rat. Horizontal bars indicate the mean value for the groups. Sm, *S. mutans*; Hp, *H. pylori*.

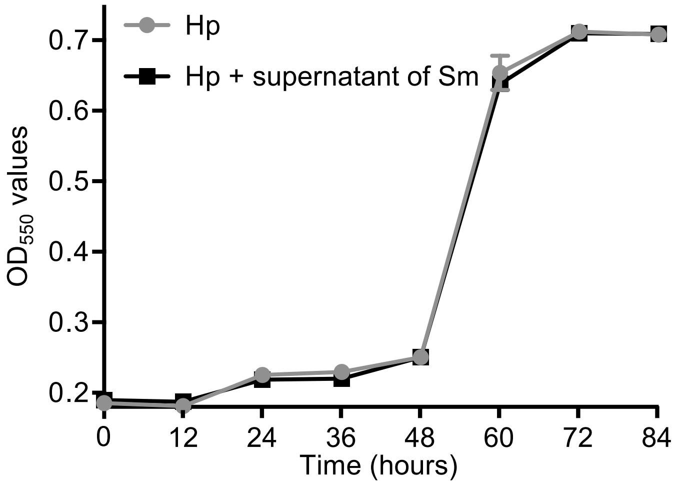

Supplementary Figure 2 Bacterial growth of *H. pylori* at multiple time points. Sm, *S. mutans*; Hp, *H. pylori*.

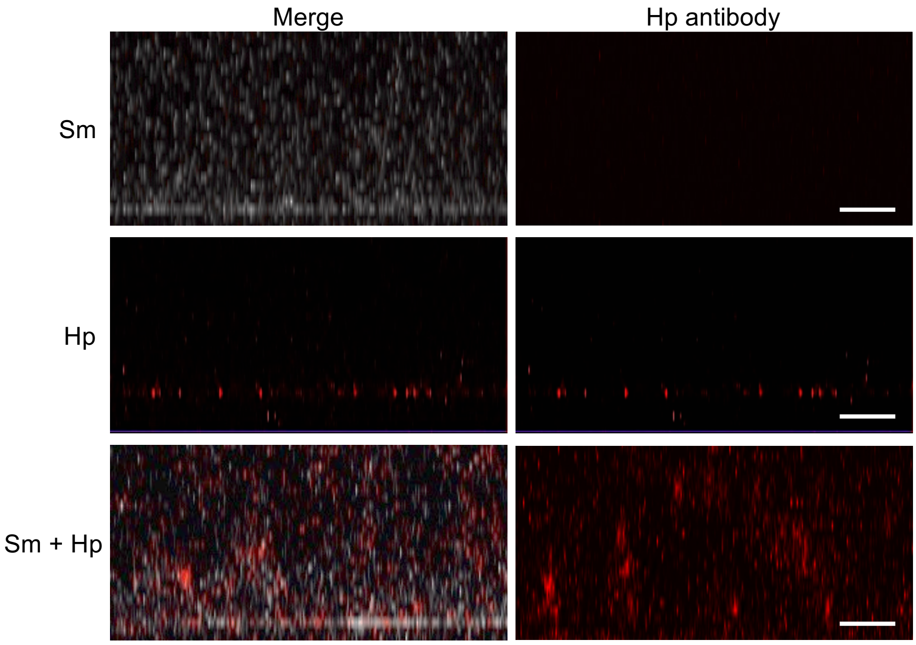

Supplementary Figure 3 Representative images of biofilm thickness, captured using confocal scanning laser microscopy. *S. mutans* and *H. pylori* cells are stained white and red, respectively. Bars = 20 μm. Sm, *S. mutans*; Hp, *H. pylori*.
